# Effects of Emotion Regulation Difficulties on the Tonic and Phasic Cardiac Autonomic Response

**DOI:** 10.1371/journal.pone.0102971

**Published:** 2014-07-23

**Authors:** Guillaume Berna, Laurent Ott, Jean-Louis Nandrino

**Affiliations:** 1 Univ Lille Nord de France, Lille, France; 2 UDL3, URECA, Villeneuve d’Ascq, France; 3 MESHS Lille Nord de France, Lille, France; University College London, United Kingdom

## Abstract

**Background:**

Emotion regulation theory aims to explain the interactions between individuals and the environment. In this context, Emotion Regulation Difficulties (ERD) disrupt the physiological component of emotions through the autonomic nervous system and are involved in several psychopathological states.

**Objective:**

We were interested in comparing the influence of a film-elicited emotion procedure on the autonomic nervous system activity of two groups with different levels of emotion regulation difficulties.

**Methods:**

A total of 63 women (undergraduate students) ranging from 18 to 27 (20.7±1.99) years old were included. Using the upper and lower quartile of a questionnaire assessing the daily difficulties in regulating emotions, two groups, one with low (LERD) and one with high (HERD) levels of emotion regulation difficulties, were constituted and studied during a film-elicited emotion procedure. Cardiac vagal activity (HF-HRV) was analyzed during three periods: baseline, film-elicited emotion, and recovery.

**Results:**

The cardiovascular results showed a decrease in HF-HRV from baseline to elicitation for both groups. Then, from elicitation to recovery, HF-HRV increased for the LERD group, whereas a low HF-HRV level persisted for the HERD group.

**Conclusions:**

The HERD group exhibited inappropriate cardiac vagal recovery after a negative emotion elicitation had ended. Cardiac vagal tone took longer to return to its initial state in the HERD group than in the LERD group. Prolonged cardiac vagal suppression might constitute an early marker of emotion regulation difficulties leading to lower cardiac vagal tone.

## Introduction

In the framework of emotion regulation (ER) studies, emotion is considered a regulator between an individual and his/her environment and also a phenomenon that needs to be regulated (for a comprehensive review, see Gross [1]). As proposed by Thompson [2], emotion regulation can be defined as “*the extrinsic and intrinsic processes responsible for monitoring, evaluating, and modifying emotional reactions, especially their intensive and temporal features, to accomplish one's goals*” (pp. 27–28). Emotion regulation refers to all processes that modify one or more aspects of emotion: situation selection, situation modification, attentional deployment, cognitive change, or response modulation [3]. These processes can be organized into two general emotion regulation strategies, antecedent-focused and response-focused, which are responsible for affective, cognitive, and social consequences [4]. From a more clinical point of view, Gratz and Roemer [5] conceptualize emotion regulation difficulties in a comprehensive and integrative way. Using conceptual and empirical studies, they describe various dimensions that compose emotion regulation: (a) awareness and understanding of emotions, (b) acceptance of emotions, (c) ability to control impulsive behaviors and behave in accordance with desired goals when experiencing negative emotions, and (d) ability to use contextually appropriate emotion regulation strategies flexibly to modulate emotional responses as desired to meet individual goals and situational demands [5]. Difficulties in regulating emotions are a central element constituting an underlying functional syndrome that may be shared by many mental disorders [6] and that can be observed both at a subjective (psychological) level [7] and at a physiological level, such as a reduced autonomic flexibility [8].

Based on Gratz and Roemer’s multidimensional conceptualization, psychometric tools such as the Difficulty in Emotion Regulation Scale (DERS; [5]) have been recently developed. DERS assesses the six dimensions developed in the model and constitutes the best-suited measuring tool for evaluating emotion regulation disorders [9]. Nevertheless, although self-reported measures have many advantages (quick to complete, no expensive equipment required, and fast processing of responses), a recurrent criticism is that they are more related to an individual's perception of his/her own abilities rather than being a reliable and valid measure of performance [10]. To overcome these obstacles, it is interesting to couple self-report questionnaires with objective measures of emotion [11], [12] and to record precise measures of emotion regulation processes in addition to a broader measure of a strategy identified by the subject [7].

Most studies on emotion using physiological indicators are based on cardiovascular and electrodermal measurements [13], [14]. Electrodermal activity (EDA) measurements are based on the recording of eccrine sweat gland secretions, which are controlled by the sympathetic nervous system. Sweating variations are sensitive markers of events that have a particular significance for individuals in terms of their emotional, novel, or attentional aspects [14]. More precisely, EDA is a good index of emotional arousal: regardless of the emotional valence of stimuli, the amplitude of electrodermal responses increases linearly as the ratings of stimuli arousal increase [15]. In addition to electrodermal measurements, several cardiovascular measurements have been used to characterize emotion regulation processes. Heart rate (HR) is still the most commonly used in the emotion literature [13], but it is considered an approximate index that is unable to convey the complexity of heart regulatory processes [16]. In fact, the sino-atrial node is dually innervated by the Autonomic Nervous System (ANS; increases in sympathetic activity increase HR, whereas increases in vagal activity decrease HR), but the sympathetic and parasympathetic branches can have antagonistic, synergistic, or independent effects [17], which rarely lead to a conclusion about the origin of the HR changes. In addition, fluctuations in the length of the interbeat interval (heart rate variability; HRV) can be used as an index of cardiac vagal output. This cardiac vagal control is one of the most rapid ways of impacting the heart, and is thought to be highly related to environmental demands [18].

More precisely, a higher tonic (resting) level and a greater magnitude of change from rest to task is a good index of cardiac vagal control, and this is thought to underlie the ability to regulate emotions and respond appropriately [19], [20]. Numerous research works and theories on emotion processes consider that individuals with a higher tonic HRV produce emotional responses that are better adjusted to environmental constraints [21]. Conversely, a lower tonic HRV is a trait marker of emotion dysregulation (for a review, see Appelhans & Luecken [22]). Although a few studies have associated a higher tonic respiratory sinus arrhythmia (RSA) with lower ratings of social competence [23], results have generally supported the assumption that a higher tonic HRV reflects a greater capacity for regulated emotional responses and self-regulation [24], [25]. For example, people with a higher tonic HRV cope better with stress [26], express greater positive emotion [27], [28], and make faster and more accurate responses in cognitive tasks involving executive function [25], [29]. In contrast, a lower tonic HRV is associated with a poorer self-regulatory capacity [30].

Moreover, as shown by Park et al. [25], compared to tonic cardiac activity, relatively little is known about the role of phasic cardiac activity in the context of self-regulation [20], [31], [32]. When people are exposed to a stressful situation or to emotion elicitation paradigms such as video clips depicting a conflict [19], [33], [34], phasic HRV suppression occurs. This is also observed when people are engaged in aversive or worrisome mental imagery [8], [35]. This decrease in HRV has been considered an autonomic response to stress [34], [36], which represents the withdrawal of cardiac vagal control and the activation of the defensive system [8]. In addition, these studies investigating suppression found a positive association between a higher tonic HRV and a greater phasic HRV suppression in response to stress [19], [33], [34]. Thus, a greater phasic HRV could reflect emotional regulation and provide a protective function against environment challenges [34], [37].

In this framework, the present study proposes to compare the cardiovascular activity of two groups of participants with different levels of emotion regulation difficulties while they experience laboratory-elicited emotion. Film clips were used as the emotion elicitation method because they are suited to the laboratory, can elicit specific emotions and offer good *emotional ecology* with regard to real-life, personalized recall or picture viewing methods [13]. EDA was recorded through Skin Conductance Level (SCL) to serve as a control measure for arousal and we presumed that there would be no difference between the groups throughout the experiment. Cardiovascular activity was studied using High Frequency changes in HRV (HF-HRV), a well-established indicator [38] that is more reliable than the LF/HF ratio [39]. We hypothesized that participants with a high level of emotion regulation difficulties would be characterized by: (a) a lower resting HRV level as indicated by lower HF-HRV level at baseline (i.e., the tonic hypothesis) and; (b) reduced cardiac vagal suppression as indicated by smaller phasic HRV differences from baseline to elicitation or elicitation to recovery periods (i.e., the phasic hypothesis).

Finally, to verify the specific role of the emotion regulation difficulties on cardiac autonomic responses, it is important to control for age since it has been shown that the development of ER is linked to neuronal maturation [40] and familial context [41]. Moreover, Anxiety and depression levels will also be examined because they are known to strongly affect ER processes [42]–[44] and to modify the cardiac autonomic response [45], [46].

## Methods

All participants were informed of the aims and procedure of the study and signed an informed consent form for participation in the study. Information on the hypotheses was given during debriefing, after the experimental session. This study was approved by an independent ethics committee (University of Lille, France) and adhered to the tenets of the Declaration of Helsinki.

### Participants

The experiment was presented to undergraduate students just before a lecture. They were asked to give their contact details to receive additional information and were told that they would receive no compensation. Thereafter, if they still wanted to participate, an appointment was made with the investigator. The sample consisted of 85 undergraduate students (recruited between the first and fifth year of their university course), the majority of whom were women (N_women_ = 70; 82.36%). Because gender influences emotional responses [47] and the number of men in the sample was not sufficient to constitute a group *per se* (N_men_ = 15; 17.64%), all men were excluded from the analyses. Other exclusion criteria were as follows: past or current cardiovascular, neurological, or psychiatric disorders and past or current pregnancy state. We also excluded individuals experiencing substance abuse or dependence (alcohol and other drugs), which is not uncommon in the student population and can affect ANS activity and emotion processing [48], [49]. Modules J and K from the Mini International Neuropsychiatric Interview (MINI) were used to assess these disorders and information on the risks as well as a specialized service were provided. The final exclusion criterion was the presence of Post-Traumatic Stress Disorder (PTSD) because the elicitation procedure uses films that could relate to traumatic events and trigger flashbacks [50]. We used module I from the MINI and found no participants suffering from PTSD.

Among the 70 participants, seven had to be excluded because of technical problems that made the physiological data unusable. The final study sample consisted of 63 normal-weight women, ranging from 18 to 27 years of age (20.89±1.93).

### Instruments

#### Emotional videos

In the present study, anger was selected from different basic emotions for several reasons: (a) it is one of the emotions most often investigated [13], (b) it is a basic emotion whose recognition is independent of cultural differences [51], [52]; (c) it is linked to interpersonal interactions, which facilitates its use in the laboratory; and (d) it engages emotion regulation strategies [53].

We first selected a video from the *FilmStim* database [54] because its elicitation properties were known. The *FilmStim* database consists of 64 film excerpts selected by 50 experts and rated on multiple dimensions by 364 undergraduate students. Ratings included the PANAS scales [55] and a 7-point scale of subjective emotional arousal [56], [57]. We selected video number 5 (code 15): a 93-sec excerpt from the film American History X [58], predominantly categorized as eliciting anger with a high arousal score (5.84/7) and the maximum negative affect score among databases (2.73/5). Although this clip has good qualities for our experiment, we wanted to add a more ecological stimulus to our video set, even if it had not been previously tested. The *FilmStim* database contains only film excerpts performed by actors, which could diminish elicitation in people with emotion regulation difficulties [59]. So, we first reviewed a dozen videos available on the web and selected two: a 69-sec video showing a real fight between two people and a 60-sec public service advertising campaign by the Department of the Environment in Northern Ireland and the Republic of Ireland's Road Safety Authority named *The Faster the Speed, the Bigger the Mess* (viewable for example: http://www.youtube.com/watch?v=Lw8GPiiOCpI). Soundtracks from all the videos were removed to focus the attention of participants on the images.

Moreover, to investigate the differences in arousal elicited by the films, a single factor (3 videos) with repeated-measures (RM) ANOVA was conducted on SCL. As the sphericity assumption was violated, the Greenhouse-Geisser correction was employed. ANOVA revealed no effect of the films on SCL (*F*(1.764, 109.392) = 0.52, *p*>.10).

#### Psychological assessment

*Emotion Regulation.* The DERS was recently validated in a French-speaking sample (DERS-F; [60]), and we used a shortened version (half the items) of this scale to avoid tiring the participants (DERS-18; [61]). DERS-F presents high internal consistency (α = .92), good test-retest reliability (ρ = .88, *p*<.01), and very good compatibility with the original version (94%). Factorial analysis reveals six factors: nonacceptance of emotional responses (Nonacceptance), difficulties engaging in goal-directed behavior (Goals), impulse control difficulties (Impulse), lack of emotional awareness (Awareness), limited access to emotion regulation strategies (Strategies), and lack of emotional clarity (Clarity). Some examples of items are: Nonacceptance: *When I’m upset, I become embarrassed for feeling that way*; Goals: *When I’m upset, I have difficulty focusing on other things*; Impulse: *When I’m upset, I lose control over my behavior*; Awareness: *I am attentive to my feelings*; Strategies: *When I’m upset, I believe that wallowing in it is all I can do*; Clarity: *I am clear about my feelings*. Participants answer on a 5-point scale ranging from 1: *almost never* to 5: *almost always*. The overall DERS-18 score ranges from 18 to 90 and, in the absence of a score threshold, analyses are conducted by comparing the values between the groups based on either the overall score or the six factors.

*Anxiety and Depression.* To assess comorbid anxiety and depression levels, we used the Hospital Anxiety and Depression Scale (HADS) [62] (validated in French [63]). This is a 14-item scale that has good psychometric properties and provides very quick assessment.

#### Physiological recording equipment

Physiological signals were acquired using a BIOPAC amplifier (MP35; BIOPAC Systems, Inc., Goletta, CA, USA). SCL was recorded using 6-mm Ag/Ag-Cl electrodes attached to the volar surfaces on the medial phalanges of the non-dominant hand, which contains fewer calluses [64], [65]. A solution of sodium chloride in a neutral base was used as an electrode paste. An electrocardiogram (ECG) was recorded using a pre-gelled electrode array placed in a Lead II configuration. The ECG attachment sites were prepared using 70% isopropyl alcohol. The respiration rate was not measured because it does not affect HRV in resting state recordings [66]. All signals were digitized in 24-bit resolution at a 1000 Hz sample rate. Subjects were asked to sit quietly, breathe spontaneously, and limit their movement during the experiment to minimize movement artifacts in the measurements.

### Procedure

During the experimental session conducted in a sound-attenuated and temperature-monitored room, subjects were seated in a comfortable chair. Film clips were presented on a 19-in monitor (placed approximately one meter from the subject) and the film order was counterbalanced between subjects. Participants were asked to complete the MINI interview and, if all inclusion criteria were met, to complete self-report questionnaires. This step took 35 to 55 min, which also served as a laboratory adaptation period. Then, physiological recording equipment was presented, set up, and the experiment was started. The experimental design consisted of three periods: a 4-min baseline followed one minute later by the film elicitation and finally a 4-min recovery. During baseline and recovery, the subjects were asked to relax and to close their eyes. They were informed by a digitized voice when they could open their eyes again. Between each film clip, a 1-min standby was observed. After the procedure, a debriefing took place and participants were encouraged to ask any questions they wished.

### Physiological data analysis

All data processing was performed off-line using a commercial software package (MATLAB R2009b, The MathWorks Inc., Natick, MA, 2000). EDA measurements were low-pass filtered by a zero-phase digital filter with a -3 dB cut-off frequency of 1 Hz. SCL was partitioned and averaged into 80-sec epochs for baseline and recovery. Regarding cardiovascular measurements, the ECG signal was band-pass filtered at 0.5 and 35 Hz with a notch filter set at 50 Hz in BIOPAC acquisition software. The R-R intervals were calculated with BIOPAC software and then corrected with a visual examination by an experimenter who was blinded to the experimental hypotheses. HR was averaged for each period. For HRV quantification, we referred to HRV guidelines [67], [68] as well as the Kubios software user’s guide [69]. The R-R intervals were first detrended with a smoothness-prior method [70] to remove the very low frequency component (<0.04 Hz). Then, a power spectral density analysis was performed using a non-parametric method (Fast-Fourrier Transform) with a high frequency band set at 0.15–0.4 Hz. HF-HRV was natural-log transformed, and two phasic HRV indexes were calculated: ΔHF-HRV_1_ = HF-HRV_elicitation_–HF-HRV_baseline_, and ΔHF-HRV_2_ = HF-HRV_elicitation_–HF-HRV_recovery_.

### Setting up of the ERD groups

The distribution of DERS-18 total scores ranged from 24 to 76 (72.2% of the maximum possible range) with an average of 41.41±10.18. In comparison, the overall DERS score obtained in the original validation (twice as many items) from a sample of undergraduate women students was 77.99±20.72. Skewness (0.82) and kurtosis (1.07) were quite large, and the normality assumption was rejected (*p*<.10). Thus, we used the upper and lower quartiles of the DERS to create two groups: (a) the Low Emotion Regulation Difficulty group (LERD): N = 18 with scores ranging from 24 to 35 (30.39±3.25); and (b) the High Emotion Regulation Difficulty group (HERD): N = 19 with scores ranging from 45 to 76 (53.53±7.70). These two groups were significantly different in terms of the overall DERS score (*p*<.001) and each factor (Nonacceptance, Goals, Impulse, Awareness, Strategies and Clarity; all *p's*≤.03).

### Statistical analysis

For SCL, RM-ANOVA was used across seven periods, which represent the three 80-sec epochs of the baseline and recovery periods together with the elicitation period. These seven periods were referred to as t_1,2,3,elicitation,5,6,7_. For the tonic hypothesis, one-way ANOVA was used to study ERD group effect on baseline HF-HRV level. For the phasic hypothesis, two analyses were performed: a one-way ANOVA to study ERD group effects on ΔHF-HRV and a complementary factorial 3×2 (Periods × ERD groups) RM-ANOVA on HF-HRV data. For all analyses, the Greenhouse-Geisser correction was employed if the sphericity assumption was violated. When useful, pairwise comparisons with Bonferroni correction were used.

Finally, in order to control the influences of age, anxiety and depression on cardiac responses, these relationships were investigated in the 63 participants by partial least squares path modeling (PLS-PM), a partial least squares approach to Structural Equation Modeling (SEM) [71], [72]. PLS-PM uses ordinary least squares, allowing models to be tested with fewer distributional assumptions and smaller samples than SEM. Moreover, PLS-PM is less sensitive to normality problems and more suitable for avoiding indeterminacy problems [73]. Regarding investigations of the age of participants, comparisons between the groups’ path models were preferred over the introduction of a pseudo latent variable containing only one Manifest Variable (MV).

PLS-PM loadings (*w*) of MVs on each latent variable (LV) are similar to principal component regression analysis loadings, and path coefficients (β) are similar to standardized beta coefficients in a classic regression analysis. The results are shown as path coefficients with their bootstrap (2000 resamples) 95% confidence interval (CI). A path coefficient is said to be significant when the bootstrap CI does not contain zero.

Assessment of the model quality relates to: (a) the adjustment of the MVs to their respective LV by examining the unidimensionality of each LV with Dillon-Goldstein’s rho (DG-rho) and cross-loading (loadings of an MV with the other LV); (b) the proportion of explained variance between LVs, with the R^2^ determination coefficient. Moreover, a global criterion to evaluate the general performance of the model is given by a pseudo Goodness of Fit index (GoF). It is usually interpreted as the prediction power of the model but there is no threshold to enable its statistical significance to be determined and no guidance about what number could be considered a good GoF value [72].

For this part, we used the plspm package [74] (version 0.4.1) available from R software [75] (version 3.0.2).

## Results

### Descriptive statistics

Table 1 presents the means, standard deviations, observed statistics, and significance tests for age, anxiety and depression scores, physiological data, and cardiac change scores for all participants and groups.

**Table 1:** Descriptive statistics for all participants and ERD groups.

			Emotion Regulation Difficulties			
		All participants (*n* = 63)	Low (*n* = 18)	High (*n* = 19)	*F*(1,35)	*p*
**Descriptive data**						
	Age	20.89 (1.93)	21.83 (2.15)	19.79 (1.13)	13.294	**0.001**
**HADS scores**	Anxiety	7.89 (2.71)	6.28 (2.3)	9.26 (2.47)	14.468	**0.001**
	Depression	2.97 (2.24)	1.72 (1.6)	4.42 (2.59)	14.347	**0.001**
**Physiological data**						
**Baseline**	SCL time 1 (µS)	7.85 (3.08)	6.99 (2.85)	7.39 (2.7)	0.197	0.660
	SCL time 2 (µS)	7.11 (3.13)	6.11 (2.76)	6.76 (2.94)	0.469	0.498
	SCL time 3 (µS)	6.44 (3.26)	5.42 (2.88)	6.19 (3.26)	0.576	0.453
	HF-HRV (ln(ms^2^))	6.71 (1.11)	6.48 (1.38)	6.43 (1.06)	0.019	0.890
	HR (bpm)	82.81 (12.04)	81.84 (12.17)	87.6 (11.38)	2.212	0.146
**Elicitation**	SCL (µS)	7.19 (3.55)	6.3 (3.47)	7.03 (3.73)	0.378	0.543
	HF-HRV (ln(ms^2^))	6.26 (0.96)	5.97 (1.17)	6.02 (0.98)	0.023	0.881
	HR (bpm)	81.49 (10.42)	80.2 (10.58)	86.81 (10.47)	3.651	0.064
**Recovery**	SCL time 1 (µS)	7.03 (3.52)	6.37 (3.6)	6.92 (3.91)	0.204	0.654
	SCL time 2 (µS)	6.58 (3.49)	5.88 (3.44)	6.48 (4.01)	0.232	0.633
	SCL time 3 (µS)	6.39 (3.59)	5.88 (3.58)	6.33 (4.25)	0.126	0.725
	HF-HRV (ln(ms^2^))	6.48 (1.09)	6.25 (1.23)	5.95 (1.01)	0.671	0.418
	HR (bpm)	83.98 (11.10)	83.56 (10.42)	90.03 (11.41)	3.237	0.081
Δ **scores**	ΔHF-HRV1	−0.45 (0.47)	−0.52 (0.5)	−0.41 (0.33)	0.629	0.433
	ΔHF-HRV2	−0.22 (0.48)	−0.29 (0.37)	0.07 (0.47)	6.397	**0.016**

Legend: HADS: Hospital Anxiety & Depression Scale; SCL: Skin Conductance Level; HF-HRV: High Frequency Heart Rate Variability; HR: Heart Rate; ΔHF-HRV1: HF-HRV(elicitation)–HF-HRV(baseline); ΔHF-HRV2 = HF-HRV(elicitation)–HF-HRV(recovery).

The HERD participants were younger (*p*<.001), and reported more anxiety (*p*<.001) and depression (*p*<.001) than the LERD participants. The HERD group exhibited a marginally significant HR increase during emotion elicitation and recovery (*p*<.10).

Regarding the experimental conditions, there was no significant difference in the room temperature between the groups (*p* = .59) that could explain SCL differences [64].

### Control measure for arousal (SCL)

**Figure 1:**
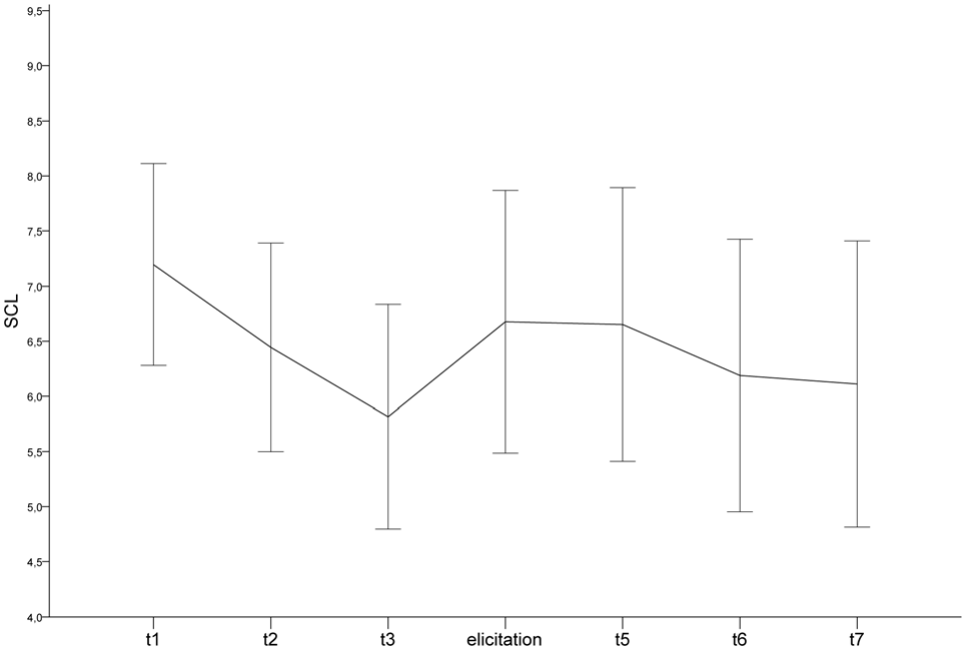
SCL for each epoch. Legend: SCL: Skin Conductance Level. Significance level indicated by the following symbols: *p<0.01, **p<0.0005. Error bars represent 95% confidence intervals.

RM-ANOVA of the EDA measurements revealed only a main effect of Period (*F*(1.612, 56.436) = 7.01, *p*<.005, η^2^ = 0.167). The main effect of ERD groups and the interaction effect of ERD groups with Period were not significant (*F*(1, 35) = 0.30, *p*>.10 and *F*(1.612, 56.436) = 0.15, *p*>.10, respectively). Mean SCLs with 95% CI for each epoch are presented in Figure 1. Follow-up pairwise comparisons revealed that SCL linearly decreased during baseline (*M*_(t2-t1)_ = −0.75, *p*<.0005; *M*_(t3-t2)_ = −0.63, *p*<.0005) and increased during emotion elicitation (*M*_(elicitation-t3)_ = 0.86, *p*<.01). Concerning recovery, it did not change significantly in the first 80-sec epoch (*M*_(t5-elicitation)_ = −0.23, *p*>.10), decreased during the second epoch (*M*_(t6-t5)_ = −0.46, *p*<.0005) and did not change in the last epoch (*M*_(t7-t6)_ = −0.076, *p*>.10).

### Tonic and phasic hypotheses

To test the tonic hypothesis, a one-way ANOVA was used to examine the effects of ERD groups on baseline level of HF-HRV: there was no significant effect (*F*(1, 35) = 0.019, *p*>.10).

**Figure 2:**
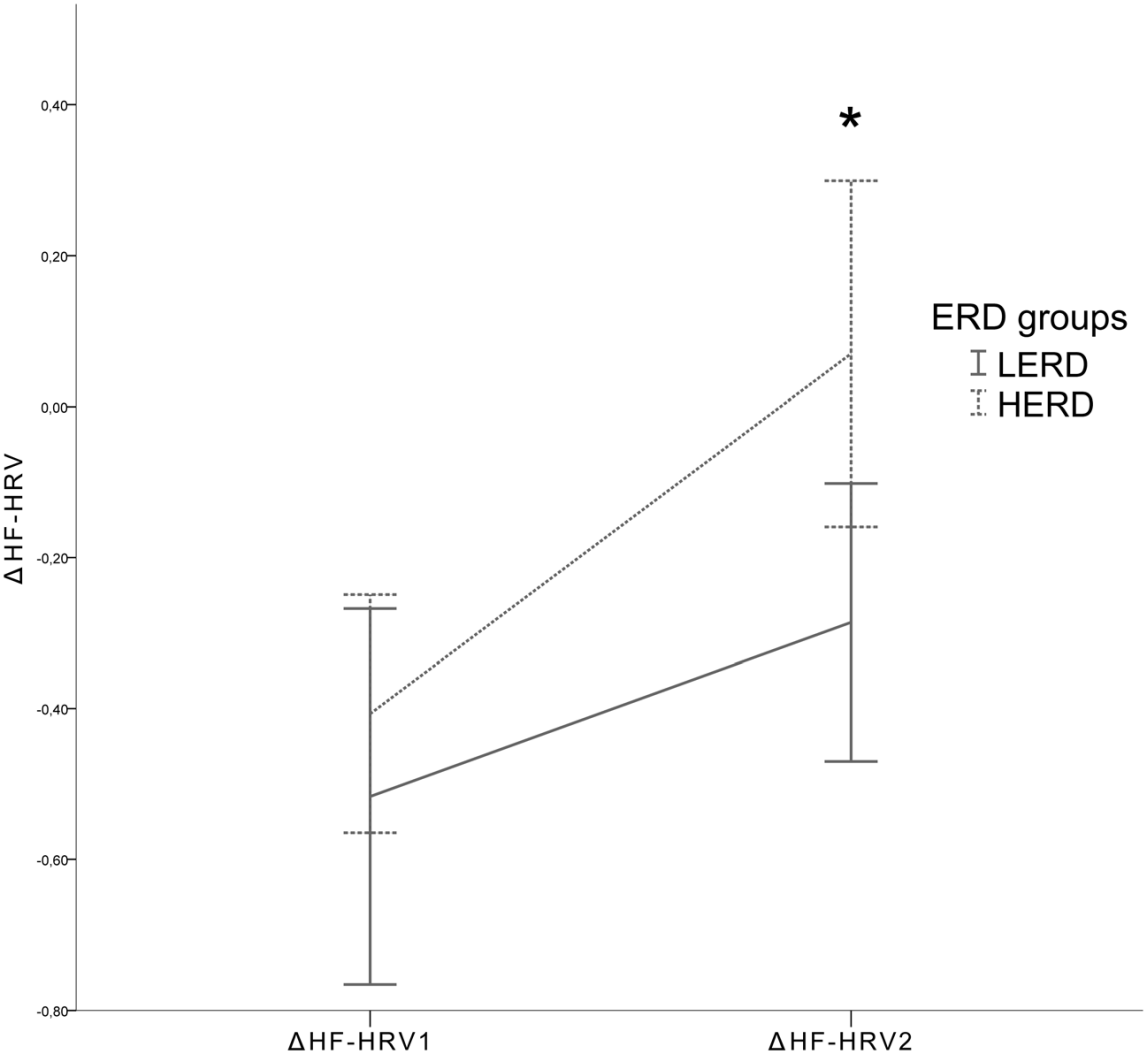
ΔHF-HRV for each ERD group. Legend: HF-HRV: High Frequency Heart Rate Variability; ΔHF-HRV1 = HF-HRV(elicitation)–HF-HRV(baseline); ΔHF-HRV2 = HF-HRV(elicitation)–HF-HRV(recovery); ERD: Emotion Regulation Difficulties; LERD: Low Emotion Regulation Difficulties group; HERD: High Emotion Regulation Difficulties group; *: Indicates significant effect of ERD groups on ΔΔHF-HRV_2_. Error bars represent 95% confidence intervals.

The same statistical analysis was used on ΔHF-HRV_1_ and ΔHF-HRV_2_ to test the phasic hypothesis. There was no significant effect of ERD groups on ΔHF-HRV_1_ (*F*(1, 35) = 0.629, *p*>.10). However, there was a significant effect of ERD groups on ΔHF-HRV_2_ (*F*(1, 35) = 6.397, *p<*.02, η^2^ = 0.155). ΔHF-HRV with 95% CI for each ERD group is presented in Figure 2.

**Figure 3:**
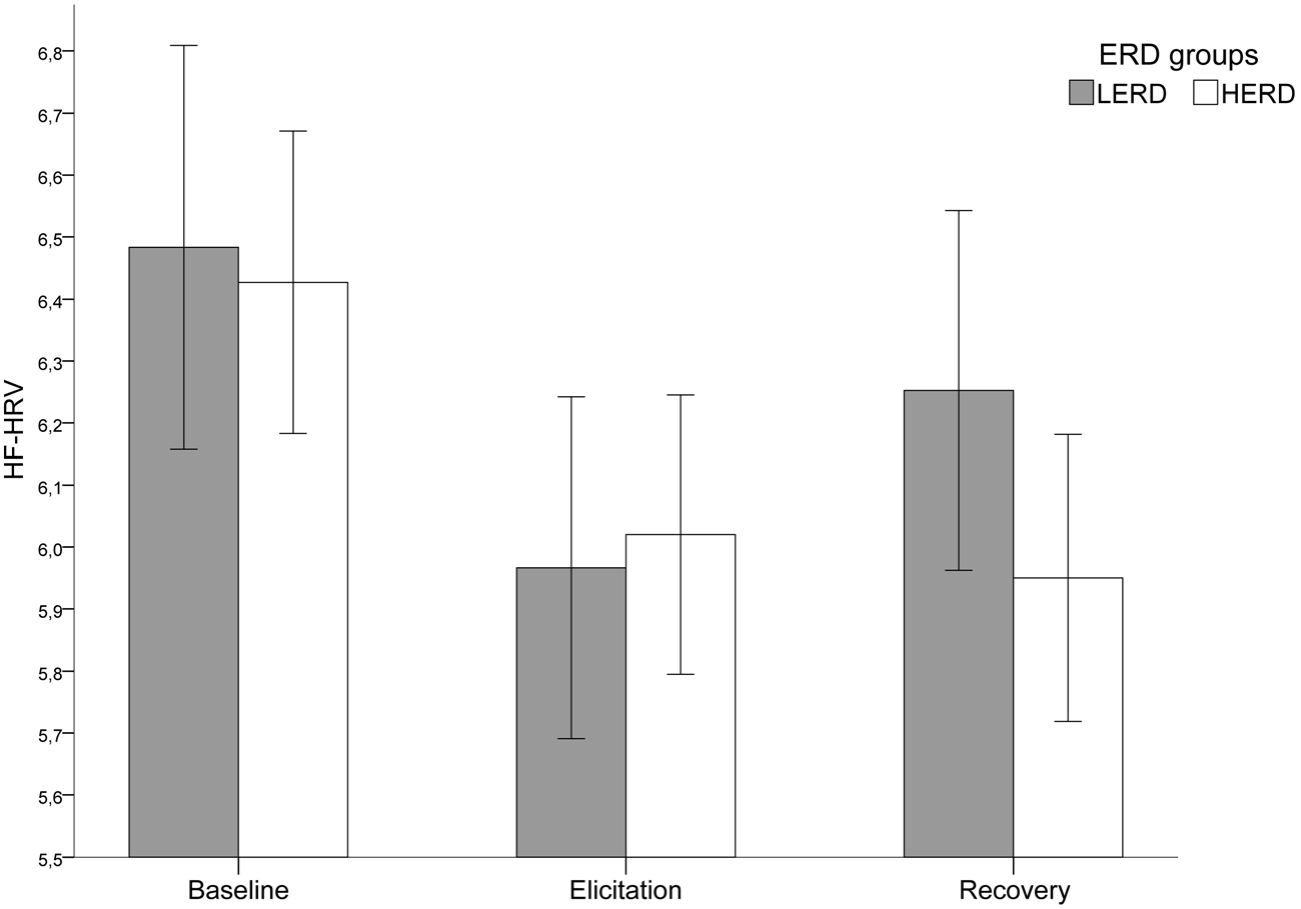
HF-HRV temporal dynamics for each ERD group. Legend: HF-HRV: High Frequency Heart Rate Variability; ERD: Emotion Regulation Difficulties; LERD: Low Emotion Regulation Difficulties group; HERD: High Emotion Regulation Difficulties group. Error bars represent standard error of means.

More precisely, RM-ANOVA was used to examine the temporal dynamics of HF-HRV between ERD groups (see Figure 3). There was a main effect of Period on HF-HRV (*F*(2, 70) = 22.08, *p*<.001, η^2^ = 0.387), no significant main effect of ERD groups (*F*(1, 35) = 0.08, *p>*.10), and a significant interaction effect between Period and ERD groups (*F*(2, 70) = 3.15, *p*<.05, η^2^ = 0.09). Pairwise comparisons revealed, for the two groups, a significant decrease in HF-HRV from baseline to elicitation: for LERD, *M*_(elicitation-baseline)_ = −0.517, *p*<.01; for HERD, *M*_(elicitation-baseline)_ = −0.407, *p*<.01). Then, for the LERD group was observed a significant increase in HF-HRV from elicitation to recovery, *M*_(recovery-elicitation)_ = 0.286, *p*<.02, which did not differ from baseline level, *M*_(recovery-baseline)_ = −0.231, *p*>.10, whereas for the HERD group, sustained low HF-HRV level was observed, *M*_(recovery-elicitation)_ = −0.07, *p*>.10.

To control the influences of age, anxiety and depression on cardiac responses, we investigated the relationships between these variables in the 63 participants. First analyses using regression models revealed multicollinearity issues, as indicated by an abnormally high Variance Inflation Factor [76] (VIF>10). To overcome this issue, a path analysis was performed using PLS-PM [77]. The model tested involved 10 MVs loaded on 3 LVs: (1) emotion regulation difficulties, (2) anxiety & depression and (3) cardiac vagal recovery. LV1 was defined with the 6 factors of the DERS (Nonacceptance, Goals, Impulse, Awareness, Strategies and Clarity), LV2 with the 2 dimensions of the HADS (Anxiety and Depression) and LV3 with ΔHF-HRV_2_ and HF-HRV recovery levels. All loadings of the outer model were satisfactory (*w*>0.65) except for DERS Nonacceptance (*w* = 0.5) and DERS Awareness (*w* = 0.4). However, to maintain the consistency of meaning of the latent variable, all observed MVs were retained. The quality of the outer model was good regarding the unidimensionality of all LVs (all DG-rho >0.79) and cross-loadings (MVs were always more correlated with their respective LV). The GoF of the model was 0.40.

The results showed that ERD was significantly linked to cardiac vagal recovery (β = −0.33, 95% bootstrap CI = [−0.64; −0.02]) and to anxiety and depression (β = 0.67, 95% bootstrap CI = [0.5; 0.81]). R^2^ determination coefficients for these relationships were 0.15 and 0.45, respectively. However, the anxiety and depression LV was not significantly linked to cardiac vagal recovery (β = −0.06, 95% bootstrap CI = [−0.36; 0.27]). Regarding the age influences, a bootstrap t-test (2000 resamples) with a centroid method (sample divided into two equal parts) revealed that no comparison of path coefficients between age groups reached significance (all *p's*>0.10).

## Discussion

The current study investigated the autonomic nervous system responses (as indicated by SCL and HF-HRV) of two groups of participants with low or high emotion regulation difficulties, as conceptualized by Gratz & Roemer [5], during a film-elicited emotion procedure. A supplementary path model analysis was run to control the effects of age, depression and anxiety on the relationship between ERD and cardiovascular measurements.

Electrodermal results showed, as presumed, that the film-elicited emotion procedure was effective and similar for both groups. Cardiovascular results showed a decrease in HF-HRV from baseline to elicitation for both groups. Then, from elicitation to recovery, the values increased and returned to baseline level for the LERD group, whereas a low HF-HRV level persisted for the HERD group. These results confirm the phasic hypothesis: participants with greater difficulties in regulating their emotions showed smaller phasic HRV differences. Importantly, this only happened during recovery: HRV suppression was prolonged and ANS responded as if the emotion was still present. In other words, people with greater difficulties in regulating their emotions took longer to recover from the emotion elicitation. These results are consistent with the Polyvagal theory of Porges [78] and the Neurovisceral Integration Model (NIM) of Thayer & Lane [30], [79]. These theories suggest that when the fast vagal modulation of cardiac function is decreased, the organism is less able to track the rapid changes in environmental demands and is less able to organize an appropriate response. Such a delay in the physiological response during a recovery period following an emotion elicitation has been observed in patients suffering from generalized anxiety disorder (GAD; [80]). The authors observed that the implementation of an emotion regulation strategy was associated with patterns of regulation that were different in control participants and GAD patients. Of particular interest, HF-HRV in GAD participants was lower than the control during the recovery period. This result and our observations support the idea that prolonged HRV suppression reflects a deficit of sustained regulation and/or a failure to habituate to emotional stimuli in HERD individuals.

However, the tonic hypothesis (lower HF-HRV level at baseline for the HERD group) was not supported by the results. As suggested in a recent meta-analysis [81], HRV is linked to various outcomes relevant to regulated emotional responding, such as the use of constructive coping strategies [82], subjective well-being [83] or the adoption of reappraisal regulation strategies [84]. One possible explanation of this discrepancy can be attributed to the age of the sample. As suggested recently [33], inadequate vagal modulation of cardiac output could be an etiological mechanism or a factor of vulnerability in a current and future psychological disorder that may emerge during adolescence. Moreover, even if genetic factors show some substantial contribution to ANS regulation, environmental factors may explain 40 to 64% of variance [85]. In this context, it is reasonable to think that the tonic level of HRV could be a consequence of prolonged and/or repeated HRV suppression associated with emotional or stressful situations. As emotion is ineffectively regulated, the HRV recovery level decreases over a longer time and the tonic HRV level diminishes gradually. In fact, it is possible that the majority of the 20-year-old undergraduate students (19.79 years old in the HERD group) have not yet experienced sufficient ineffective emotional regulation to express a lowered tonic HRV. Thus, participants from the HERD group would be a subclinical group, or at least a group that is highly vulnerable to the development of a psychological disorder. Furthermore, the depression and anxiety scores of the HERD group support this idea and are discussed in the next paragraph. Another argument in favor of an effect of prolonged cardiac vagal recovery on tonic HRV level can be found in vagally mediated therapeutic techniques such as HRV biofeedback, relaxation, meditation or mindfulness. One of the goals of these techniques is to promote parasympathetic influences at rest that are characteristic of a physiological state of well-being. By increasing the frequency and duration of a high tonic HRV level, one can compensate for the physiological pattern we reported in HERD [86]. These results need to be supplemented by a longitudinal study in which the predictive effects of the reported physiological pattern (prolonged HRV suppression/reduced recovery level without differences in tonic HRV level) on vulnerability to psychological disorders can be investigated.

As previously mentioned, the HERD group had higher scores of anxiety and depression in comparison to the LERD group and there was a small effect of the interaction between ERD groups and Period (η^2^ = 0.09), so analyses were conducted to control these effects. The results of PLS-PM revealed that cardiac vagal recovery was only linked to ERD (β = −0.33) and anxiety and depression did not play a mediating role in this relationship (β = −0.06). Moreover, anxiety and depression were only linked to ERD (β = 0.67) with the usually observed explained variance (R^2^ = 0.45) [87], [88]. Finally, age did not seem to play a role in these relationships in the sample of 63 young participants.

Regarding the HAD scale, it was not designed for diagnostic purposes but can be used in a symptomatic approach and provides cut-off scores on each sub-scale [89]: 10 with a specificity goal and 8 with a sensitivity goal. Thus, the HERD group displayed an anxious state only at a moderate level (mean score = 9.26). Moreover, it has been previously reported that the tonic HRV level is reduced in depression [45], independently of a prior history of cardiovascular disease, and that patients with depression and comorbid GAD display the greatest reductions in tonic HRV [46], [90]. As mentioned above, we did not observe significant tonic HRV differences between the LERD and HERD groups (*p*>.10).

Taken together, these results highlight a specific effect of difficulties in regulating emotion on the cardiac autonomic response, which is independent of age or level of anxiety and depression, and strengthens the suggestion that the HERD group is a subclinical group or one highly vulnerable to psychological disorders.

### Limitations and broader perspectives

As previously discussed, the mean age of our study groups was quite low, and it is possible that there is an age effect on the physiological responses of emotional processing, which could limit the generalization of these results. Therefore, future studies are needed to determine the relationship between the development of emotion regulation processes and autonomic cardiac regulation during childhood, adolescence, or in aging. Another limitation involves the sex of our participants: as previously stated, gender influences emotional processes and responses [47]. It is important to examine whether men use the same physiological regulation mechanisms as women. In this study, the group of men was too small to be studied *per se* but we will include men in future studies.

In addition, in the LERD group, the results showed that HF-HRV in recovery was higher than during emotion elicitation but lower than during baseline. It is important to verify that, with a longer time of observation after emotion induction, the cardiac signal during the recovery phase would completely return to the baseline level.

Moreover, the emotion elicitors used in this study were film clips that were selected specifically to elicit anger. Although we chose the stimuli carefully, it is possible that they do not fully account for emotional regulation mechanisms that occur in everyday life. Although using a video as an emotion elicitor is a good way to compare participants, and is certainly more similar to everyday life than still pictures used in some experiments, their *emotional ecology* is diminished due to the actors’ performance or to the passive state of the subject. Several research methods have been developed to overcome this limitation: (a) by making the stimulus more realistic using 3D material [91] or by using more immersive procedures such as virtual reality [92] (example here: http://www.dukescience.org/content/studies/behavioralmeasures), and (b) by performing daily measurements using portable devices (smartphones, tablets) that can be coupled with physiological recordings. These innovative techniques offer new ways of exploring and understanding the physiological mechanisms involved in the regulation of emotions experienced *in situ* by the participants. In addition, we only used anger elicitors and we need to verify whether the same pattern is observed with other negative emotions and also with positive psychological states.

### Conclusions

The cardiac autonomic pattern is influenced by emotion regulation difficulties as conceptualized by Gratz & Roemer. People who experience emotion regulation difficulties exhibit inappropriate cardiac vagal recovery after a negative emotion ends. This may reflect an early indicator of vulnerability to psychological disorders or cardiovascular diseases [21], [81], [93], [94]. However, further investigations are needed to clarify the influences of age, gender, type and valence of emotions, and more ecological situations on the relationships between difficulties in regulating emotions and cardiac functioning.
